# 3D Powder Printed Bioglass and β-Tricalcium Phosphate Bone Scaffolds

**DOI:** 10.3390/ma11010013

**Published:** 2017-12-22

**Authors:** Michael Seidenstuecker, Laura Kerr, Anke Bernstein, Hermann O. Mayr, Norbert P. Suedkamp, Rainer Gadow, Peter Krieg, Sergio Hernandez Latorre, Ralf Thomann, Frank Syrowatka, Steffen Esslinger

**Affiliations:** 1 Department of Orthopedics and Trauma Surgery, Medical Center—Albert-Ludwigs-University of Freiburg, Faculty of Medicine, Albert-Ludwigs-University of Freiburg, Hugstetter Str. 55, 79106 Freiburg, Germany; laurak1@hotmail.co.uk (L.K.); anke.bernstein@uniklinik-freiburg.de (A.B.); hermann.mayr@uniklinik-freiburg.de (H.O.M.); norbert.suedkamp@uniklinik-freiburg.de (N.P.S.); sergio.latorre@uniklinik-freiburg.de (S.H.L.); 2 School of Engineering, James Watt South Building, University of Glasgow, Glasgow G12 8QQ, UK; 3 Institute for Manufacturing Technologies of Ceramic Components and Composites, University of Stuttgart, Allmandring 7b, D-70569 Stuttgart, Germany; Rainer.Gadow@ifkb.uni-stuttgart.de (R.G.); peter.krieg@ifkb.uni-stuttgart.de (P.K.); 4 FMF—Freiburg Materials Research Center, University of Freiburg, Stefan-Meier-Str. 21, D-79104 Freiburg, Germany; ralf.thomann@fmf.uni-freiburg.de; 5 Interdisciplinary Center of Materials Science (CMAT), Martin Luther University Halle, Heinrich Damerow Str. 4, D-06120 Halle, Germany; syrowatka@cmat.uni-halle.de

**Keywords:** 3D printing, bone scaffolds, biocompatibility in vitro, bioglass, β-TCP

## Abstract

The use of both bioglass (BG) and β tricalcium phosphate (β-TCP) for bone replacement applications has been studied extensively due to the materials’ high biocompatibility and ability to resorb when implanted in the body. 3D printing has been explored as a fast and versatile technique for the fabrication of porous bone scaffolds. This project investigates the effects of using different combinations of a composite BG and β-TCP powder for 3D printing of porous bone scaffolds. Porous 3D powder printed bone scaffolds of BG, β-TCP, 50/50 BG/β-TCP and 70/30 BG/β-TCP compositions were subject to a variety of characterization and biocompatibility tests. The porosity characteristics, surface roughness, mechanical strength, viability for cell proliferation, material cytotoxicity and in vitro bioactivity were assessed. The results show that the scaffolds can support osteoblast-like MG-63 cells growth both on the surface of and within the scaffold material and do not show alarming cytotoxicity; the porosity and surface characteristics of the scaffolds are appropriate. Of the two tested composite materials, the 70/30 BG/β-TCP scaffold proved to be superior in terms of biocompatibility and mechanical strength. The mechanical strength of the scaffolds makes them unsuitable for load bearing applications. However, they can be useful for other applications such as bone fillers.

## 1. Introduction

To tackle the complications associated with autograft and allograft methods, bone graft substitutes, known as bone scaffolds, were developed. Various artificial materials have been used to bridge bone defects and these are discussed below. Synthetic bone scaffolds only possess, at most, two of the four ideal graft material characteristics; osteointegration and osteoconduction [1]. However, there are a wide variety of challenges relating to the development of a successful graft material. According to Bose et al. [2], there are four key requirements for an “ideal” bone scaffold. Biocompatibility: The scaffold should be able to support normal cellular activity without inducing any local and systemic toxicity in the host tissue. Mechanical Properties: The mechanical properties of a bone scaffold should be similar to that of the host tissue it is replacing. It is also important that the modulus of elasticity is similar. Pore size: Scaffolds must have interconnected porosity for the supply of essential nutrients and oxygen, as well as bone tissue ingrowth. Bioresorbability: Scaffolds should be able to degrade in vivo, ideally at a rate similar to that of new tissue growth.

Most other literature agrees on these requirements [3,4], while Polo-Corrales et al. [5] also advocate the importance of incorporating growth factors into the scaffolds by methods such as controlled release of injection to enhance bone growth. 

Calcium phosphates are both osteointegrative and osteoconductive and form a hydroxyapatite (HA) layer when implanted into the body. The main advantage of using calcium phosphates is that they are highly biocompatible and there are no reports of systemic toxicity or foreign body reactions following their implantation [1,6]. The degradability of ceramics is determined, inter alia, on water’s solubility. The calcium to phosphate ratio is crucial in this regard. HA has a Ca/P ratio of 1.67. Other calcium phosphate ceramics such as β-tricalcium phosphate (β-TCP) have a more favorable 1.5 ratio and thus faster degradability [7,8]. Bioglass (BG) contains minerals which occur in the body and the quantity of calcium and phosphorous oxides present are similar to that of natural bone [9]. When BG comes into contact with body fluid, it forms a HA-like layer on its surface. The layer has a similar mineral composition to that of natural bone and this allows the BG to form a strong bond with surrounding biological tissue [10,11]. It has been used to form coatings on metal implants which improve osteointegration and to fill voids in bone grafts [1]. BG [9] and β TCP [12] were both used as bone replacement materials. These are the materials explored in this project.

3D powder printing was developed by Michal J. Cima and co-workers [13] in 1993, based on the conventional inkjet printing mechanism. It is an additive manufacturing method used to print the data from a CAD model. The model is printed in layers, composed from a starch-based powder bed and glued together with a liquid binder [14]. The technique allows for close control in the fabrication of complex 3D geometries [15]. A 3D CAD file is converted into cross sections between 0.01 and 0.2 cm thick and these cross sections are printed as slices layered on top of each other from the bottom of the model to the top. 

One particularly well developed use for 3D printing in medicine is bone replacement. Bone scaffolds can be fabricated by a variety of methods, including chemical or gas foaming, solvent casting, freeze drying and thermally induced phase separation [16,17]. Due to the ability to precisely control geometry and porosity, it is possible to create an implantable material with bone-like characteristics [14]. Much of the literature on the subject agrees that 3D printing is superior to these other methods, primarily due to the control over pore size, shape and interconnectivity which it provides [18]. Because of the high variability between individual bone defects, the customization of models allowed by 3D printing is also particularly advantageous in bone scaffold applications for creating implants specific to a particular defect. Another advantage of using 3D printing to create bone scaffolds is the wide range of materials which can be used with this fabrication method; it allows for the use of both polymers and ceramics, as well as composite combinations of these materials [19]. According to Butscher et al. [20], the only limitation for the use of a material is its availability in powder form. This is obviously in reference to the ability of the printer to create the scaffold geometry, and does not consider the scaffold’s ability to act as an efficient bone replacement, but it demonstrates the diversity of 3D printing regardless. Butscher et al. [20] states that the geometries which can be created are only restricted by the resolution of the 3D printing. The resolution depends mostly on the layer thickness, which is ultimately controlled by the properties of the powder itself. Each different material in powder form has a different particle size, and this impacts the printing process parameters and the binder required. Furthermore, the powder particle size impacts upon the mechanical strength of the scaffold, as particle size has a direct influence on porosity, and mechanical strength is directly influenced by porosity [21]. The porosity can be decreased, and therefore the mechanical strength increased, by using an optimized combination of different particle sizes [22]. In addition to the powder used, the printing process itself also affects the mechanical properties of the scaffolds produced. The mechanical properties of scaffolds created by 3D printing can depend largely on the structural and process parameters, rather than purely on the properties of the materials used [23]. The material’s mechanical properties can be significantly altered following printing process due to complex interaction events. Significant factors in the mechanical strength of printed parts include the concentration of binder used and the layer orientation during printing [24,25]. According to Chumnanklang et al. [24]; the higher the binder concentration the higher the mechanical strength of the printed part will be. In addition, Vlasea et al. [25] explores the effect of layer orientation during printing on the mechanical properties of the part. The Aim of this study was to explore to what extent the fabrication technique for a biphasic composite out of BG and β-TCP is appropriate for development of scaffolds for use in biological applications, like bone regeneration.

## 2. Results

### 2.1. Sample Characterization

**Figure 1:**
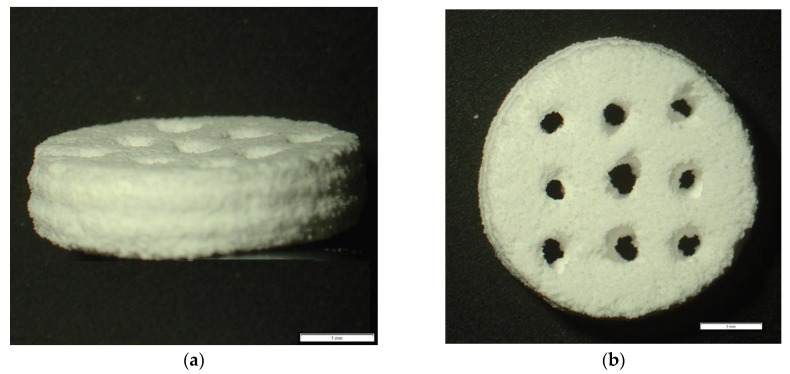
Side view (**a**); and top view (**b**) of 3D printed scaffold. The scale bar is 1 mm. Images were taken with Olympus (Tokyo, Japan) SZ-61.

The diameter and height of the scaffolds were measured using Vernier calipers. The mean diameters were calculated to be 10.19 ± 0.06 mm for BG, 10.41 ± 0.25 mm for β-TCP, 11.09 ± 0.02 mm for the 50/50 and 10.60 ± 0.02 mm for 70/30 scaffolds. This difference was deemed to be statistically significant (*p* < 0.05). The mean heights were calculated to be 2.88 ± 0.1 mm for BG, 2.45 ± 0.16 mm for β-TCP, 2.84 ± 0.12 mm for the 50/50 scaffolds and 2.94 ± 0.26 mm for the 70/30 scaffolds; a small difference which was considered not to be statistically significant (*p* > 0.05). Figure 1 shows different views of the scaffolds captured under a stereo microscope. A statistically significant difference in the weight of the materials was detected (*p* < 0.05). The mean weight was calculated to be 251.92 ± 12.9 mg for BG, 190.3 ± 7.13 mg for β-TCP, 223.85 ± 15.56 mg for the 50/50 scaffolds and 238.67 ± 18.91 mg for the 70/30 scaffolds. The dimensions of the 3D printed Samples are summarized in Table 1.

#### 2.1.1. EDX Analysis

**Figure 2:**
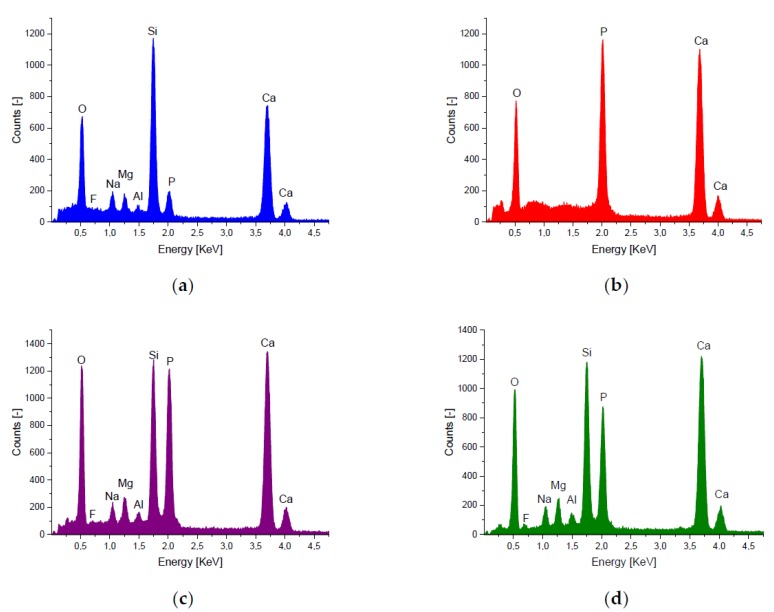
EDX Spectra of 3D printed Scaffolds: (**a**) BG; (**b**) β-TCP; (**c**) 50/50; and (**d**) 70/30; performed as area scan (area 0.81 mm^2^).

The spectrum produced from EDX analysis of the BG showed the presence of oxygen (O), silicon (Si), calcium (Ca), phosphorous (P) and small traces of sodium (Na), magnesium (Mg), aluminum (Al) and fluorine (F). The composites showed the same elements but in different compositions. The spectra are shown in Figure 2. The mean mass percentage of each element present in the tested materials is shown in Table 2. In addition, the mean Ca/P ratio for each material was calculated. The mean Ca/P ratio for 50/50 was calculated as 1.9. For 70/30, the mean Ca/P ratio was calculated to be 2.0. Compared with those results, the β-TCP spectrum showed only Ca, O, P with Ca/P ratio of 1.5. The pure BG showed a Ca/P ratio of 5.3, as expected for a 45S5 BG. The elemental compositions of all samples are summarized in Table 2.

2.1.2. 3D Laser Scanning

**Figure 3:**
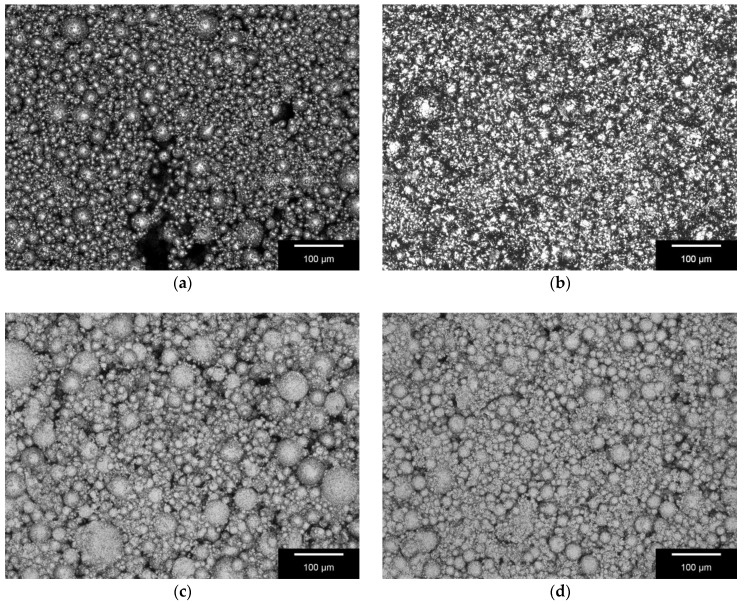
3D Laserscanning Image of Samples Surface; Differences in the particle size of the used granules can be seen, as well as the presence of only a few sinter bridges: (**a**) BG; (**b**) β-TCP; (**c**) 50/50; and (**d**) 70/30. Scale bar = 100 µm. Images were taken with Keyence VK-X210 (Keyence, Osaka, Japan). Magnification, 400×.

The mean maximum surface profile height (Rz) and mean roughness (Ra) for each material type are shown in Table 3, along with the standard deviation and percentage relative standard deviation for each measurement. Figure 3 shows the surface roughness of the four different samples.

**Figure 4:**
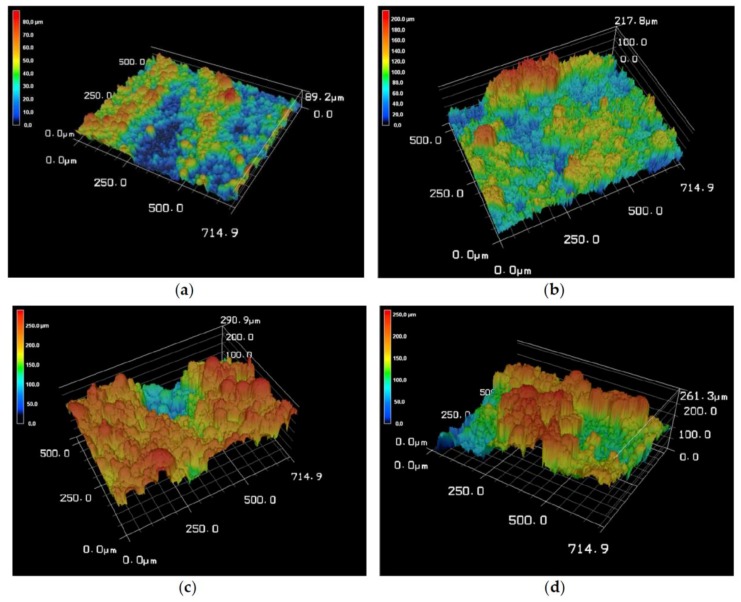
3D reconstruction of Surface of composites: depending on the different composition and particle size of used granules, a different surface roughness can be observed: (**a**) BG; (**b**) β-TCP; (**c**) 50/50; and (**d**) 70/30. Images were taken with Keyence VK-X210. Magnification, 400×.

The 3D scaffolds made of the pure substance BG showed the smallest roughness, Ra 11.82 ± 1.58 µm for BG vs. 28.92 ± 4.71 µm or 27.61 ± 8.86 µm for the composites. Compared to BG, β-TCP showed a higher roughness (Ra) of 26.08. Figure 4 shows the variation in surface roughness on different areas of the scaffolds via 3D reconstructions of the scaffold surfaces. The highest peaks, shown in red, are in the 200–300 µm range. There is a significant difference between the mean values of Ra and (*p* = 0.05) as well as a significant difference between the mean values of Rz (*p* = 0.05). 

#### 2.1.3. Mercury Porosimetry

**Figure 5:**
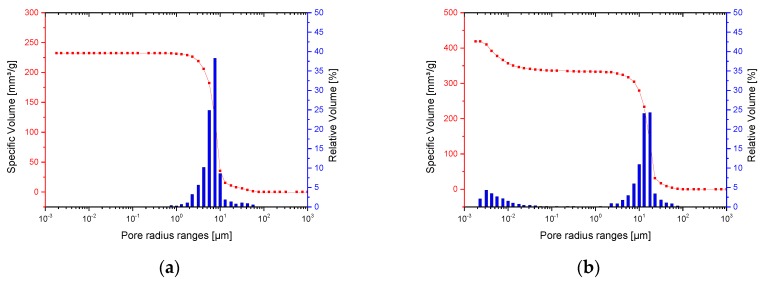

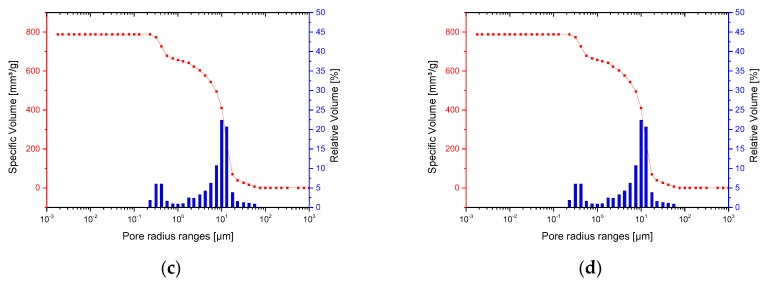
Pore size distribution: (**a**) pure BG; (**b**) pure β-TCP; (**c**) 50/50; and (**d**) 70/30. Because of the different particle sizes and milling grades of the used granules, different pore size distributions can be observed.

The pore size distribution for β-TCP, BG, 50/50 and 70/30 materials are shown in Figure 5. The measurements were similar for all materials and show that the majority of pores present are macropores with diameter 1–15 µm. The results also demonstrate the presence of some larger macropores with diameters between 15 and 60 µm, along with some mesopores (2–100 nm) and micropores (<2 nm) at the opposite end of the scale for β-TCP. Measurements of other parameters obtained from the mercury porosimetry analysis are outlined in Table 4. The average pore radius was between 5.80 and 12 µm. Total porosity was in the region of 46–75%.

#### 2.1.4. Compression Test

**Figure 6:**
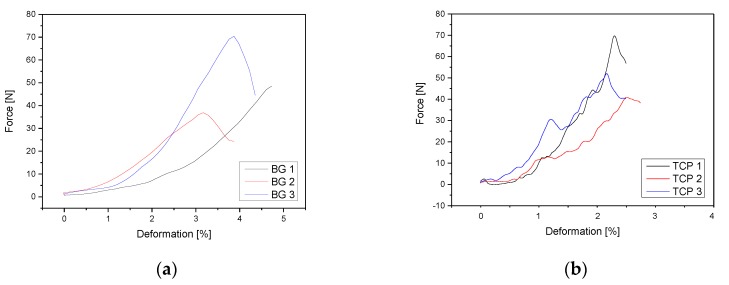

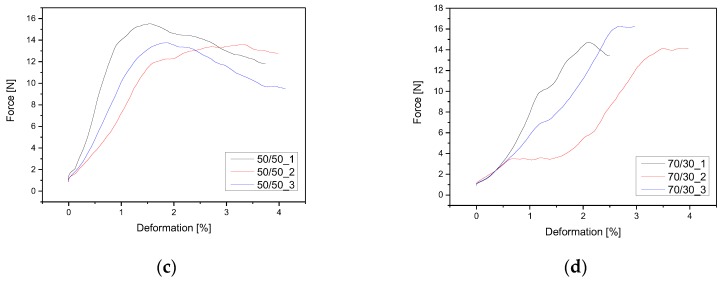
Force-Deformation curves: (**a**) pure BG; (**b**) pure β-TCP; (**c**) 50/50; and (**d**) 70/30. Experiments performed at Zwick Roell Universal Testing machine Z005 using Test Xpert II Software (*n* = 3).

The results of the compression tests for the BG, β-TCP, 50/50 and 70/30 scaffolds are shown in Table 5. The original plot, produced by the Zwick “Test Expert II” software (Zwick Roell Group, Ulm, Germany), reported a maximum force higher than the true maximum force that the material could withstand. This was due to the presence of the large pore channels in the scaffolds: when the powder structure was compressed, the large pores closed, thus forming a more solid structure that provided greater resistance to the applied force. For this reason, the first peak in the resultant plot was taken to be the maximum force which the scaffold could withstand. Figure 6 shows the Force-Deformation curves for the different samples.

The mean compression strength was calculated to be: 0.64 ± 0.21 MPa for BG; 0.64 ± 0.17 MPa for β-TCP; 0.15 ± 0.01 MPa for the 50/50 composition; and 0.17 ± 0.01 MPa for the 70/30 scaffolds. A comparison of failure load and compressive strength of the different 3D printed scaffolds is shown in Table 5. There is a significant difference between the mean failure load for all different samples (*p* = 0.05). Additionally, there is a significant difference between the mean compressive strength of the four different samples (*p* = 0.05).

### 2.2. Scaffold Biocompatibility

#### 2.2.1. Live/Dead Assay

**Figure 7:**
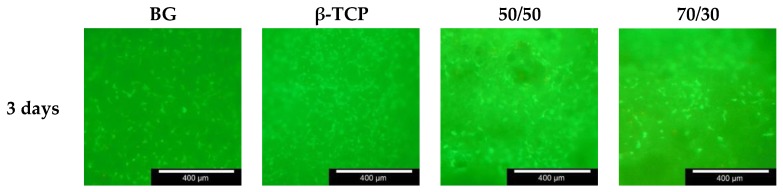

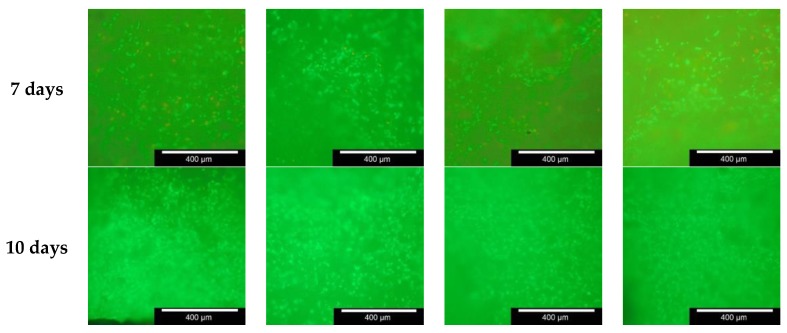
Live/dead assay images of the inner surface of the scaffolds, captured with Olympus BX 51 fluorescence microscope, broadband blue excitation with interference band-eliminator filter U-MWIB3: excitation filter Olympus BP460-495, beam splitter Olympus DM505, band-eliminator filter BA510F; living cells, green; dead cells, red. The scale bar is 400 μm.

The results from the live/dead assay, shown in Table 6, illustrate the quantity of live and dead cells present at 3, 7 and 10 days following seeding. Live cells appear luminous green and dead cells appear red. The areas with the highest visible concentration of live cells from each scaffold are shown in the images. It is clear from the images that live cells are present on the pure substances, as well as on the composite materials at 3, 7 and 10 days. There is a notable increase in the quantity of live cells present as the period increases, as more luminous green spots can be identified, which is an indication that cells are able to grow and multiply on the materials. Comparing the 50/50 and 70/30 compositions and the samples of pure BG and β-TCP, more live cells are present on the BG or 70/30 composition than the TCP or 50/50 composition. However, after 10 days, nearly the same quantity of living cells could be found on each different sample. In addition, the concentration of live cells is higher around the openings to the large porous channels in the scaffolds, which appear black in the images. Very few dead cells are identifiable in these images (see Table 6 or Figure 7). 

#### 2.2.2. Cell Proliferation Assay

**Figure 8:**
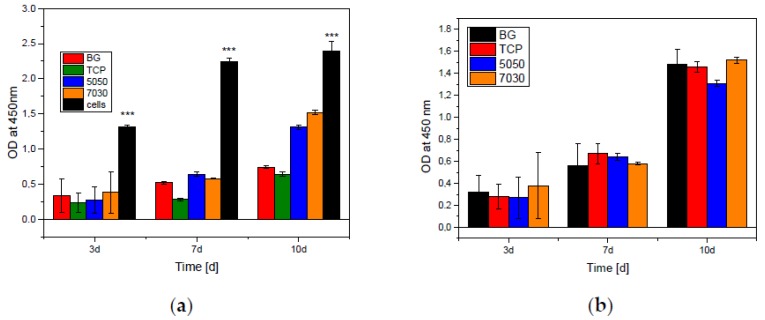
Differences in WST-1 Assay of MG-63 cells after cultivation on the different samples after 3, 7 and 10 days. Cell culture grade plastic was used as a control. Significant difference *** (*p* = 0.001). (**a**) Cells on the Scaffolds, after removing it out of the cell culture plates; and (**b**) cells remaining in the same cell culture plates, which were moving through the pores of the 3D printed scaffolds and settled at the walls of the cell culture plate (*n* = 30).

The WST-1 assay was divided into scaffold and remaining cells: Figure 8a shows the proliferation of the cells on the 3D printed scaffolds after removing them from the well plate used in the assay. Figure 8b shows the proliferation of the cells that remained in the well plate, which were moving through the pores of the 3D printed scaffolds and settled at the walls of the cell culture plate. As shown in Figure 8a, there was no significant difference in cell viability among composites after three and seven days and of cultivation. After seven days of incubation, MG 63 cells had a higher metabolic activity than at three days, which can be ascribed to the increased proliferation of the cells. Each experiment showed higher absorbance for the 70/30 scaffolds than for the 50/50 scaffolds at all time points. These results correspond very well with the measuring of total specific area. 

#### 2.2.3. LDH Assay

**Figure 9:**
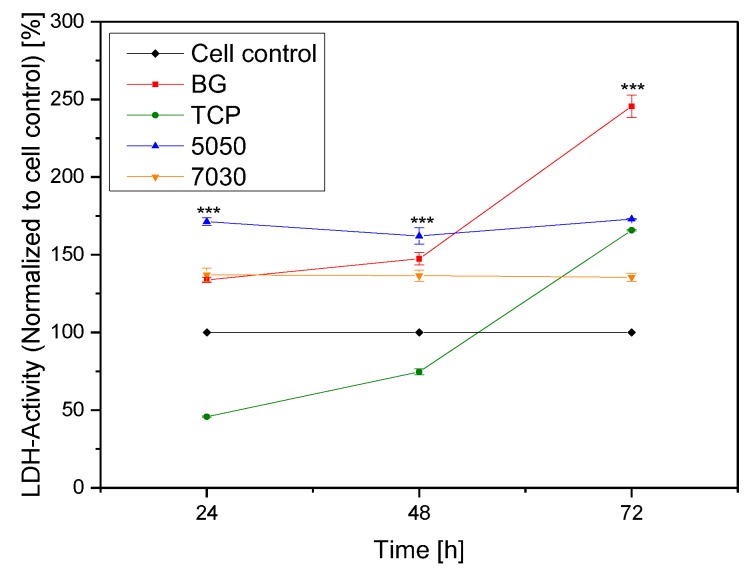
LDH Activity of MG-63 cells on the samples measured after 24, 48 and 72 h using LDH (normalized to cell control). Significant difference *** (*p* = 0.001), (*n* = 30).

The results of the Lactate dehydrogenase (LDH) absorbance, measured at 24, 48 and 72 h following seeding, are shown in Figure 9. All measured values were normalized to the cell control. Compared to all other samples the 70/30 showed the lowest LDH activity over time. The composites showed more stable LDH activity than the scaffolds made of pure substances. The LDH activity was higher for the 50/50 scaffolds than the 70/30 scaffolds in all experiments. The β-TCP showed the lowest LDH activity after 24 h, but this value increased to the value of the 50/50 after three days. BG showed a similar result, but with a shifted curve; with the highest LDH activity after 72 h.

#### 2.2.4. Immersion in SBF

**Figure 10:**
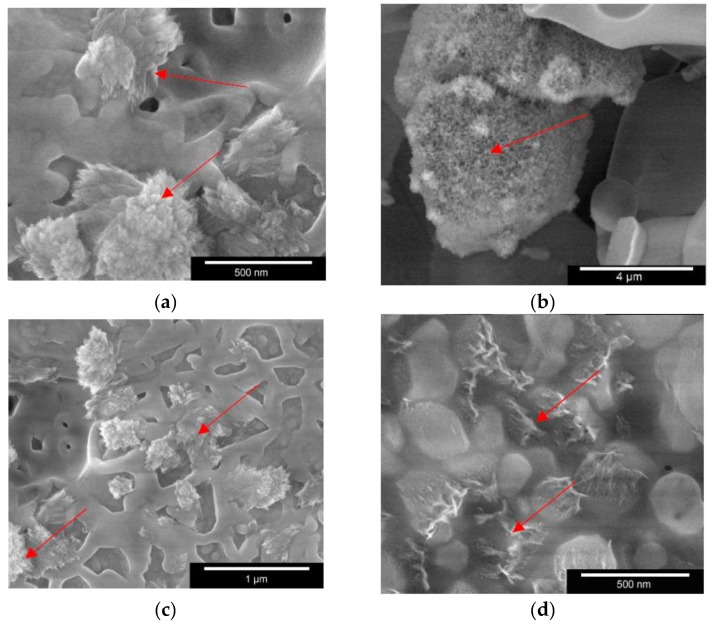
Low-vac SEM images showing crystal formation on scaffold surfaces: (**a**) BG; (**b**) β-TCP; (**c**) 50/50; and (**d**) 70/30. Red arrows indicate the HA crystals after immersion in SBF for 28 days. Images were taken with FEI Quanta 250 FEG, Large Field Detector, 5 kV, 100 Pa.

**Figure A1:**
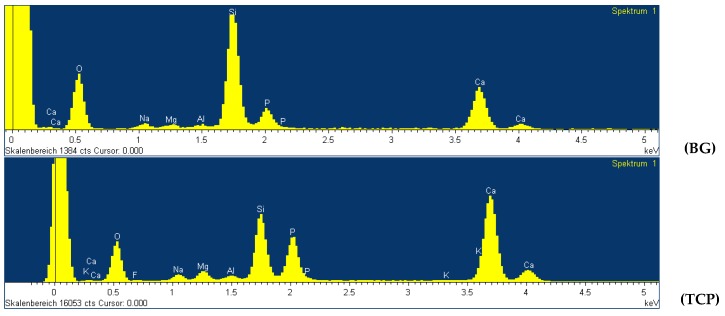

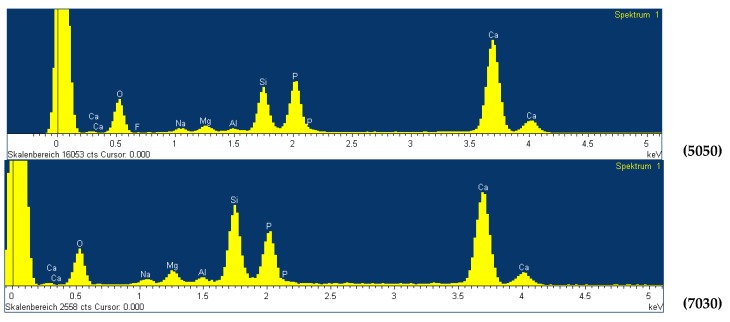
EDX Spectrum of the Nanocrystals (from top to bottom: BG, TCP, 50/50 and 70/30): Spectrum taken with FEI Quanta 250 FEG with Oxford EDX unit, at 10 kV, 100 Pa, 10 min livetime; BG: Ca/P ratio = 1.69 seems to be HA. TCP: Ca/P ratio = 1.70 seems to be HA; 50/50: Ca/P ratio = 1.72 seems to be HA; and 70/30: Ca/P ratio = 1.65 seems to be HA. The tiny structures of the Crystals in the background are shining, which is why Na, Mg, Al and Si were found in the spectra.

ESEM images of the scaffold surfaces after immersion in SBF for 28 days resulted in formation of crystals. Figure 10a shows the crystal formation on the surface of pure BG (red arrows) and Figure 10b on β-TCP. The crystal formation on BG is especially interesting due to the formation of small nano sized crystals and larger crystals after immersion in SBF for 28 days. β-TCP did not show such big crystals, because of its different solubility. Only small (nano-sized) crystals could be found. On the 50/50 scaffold (Figure 10c), long, jagged crystals, as on the BG, are visible on the material; however, apatite nanocrystals could not be observed at higher magnification. An elementary analysis via EDX (Figure A1 in Appendix) showed that those crystals in Figure 10c could be HA. Ciobanu et al. [26] reported similar HA crystals on titanium surfaces. Figure 10d shows crystal formation on the surface of a 70/30 scaffold with increasing magnification. Apatite nanocrystals could be observed in this image, as pointed by the red arrows.

After immersion in SBF for 28 days, changes in diameter, height and weight of the samples could be observed. The highest changings in weight could be observed for the composites: 12.03 ± 3.63% for 50/50 and 13.67 ± 4.91% for 70/30 (see Table 7). For the composites, changes in diameter (1.98 ± 0.25% for 50/50 and 2.36 ± 0.56% for 70/30) were also found. Smaller changes in height (0.12 ± 0.02% for 50/50 and 0.24 ± 0.12% for 70/30) were measured.

## 3. Discussion

A difference in weight was expected due to the difference in composition and the results reflect this; although the density of bioglass is more than 10% lower than the density of β-TCP, the scaffolds with a higher bioglass content were ~15% heavier than those with equal BG/β-TCP content. This is a result of the mass ratio of the mixed powder, where the fine fraction is as high as the coarse one. This leads to worse flowability, but also higher green density of the printed specimen. Although the scaffolds were printed with identical dimensions, the mean diameter of the 50/50 scaffolds was ~5% larger than the mean diameter of the 70/30 scaffolds. This difference is likely a result of the different powder compositions used in the printing process. Vorndran et al. [27] demonstrated that grain size can affect particle interconnectivity and sintering behavior. Based on this study, it is possible that larger grains were present in the 50/50 powder composition, which may have resulted in scaffolds with poor particle interconnectivity. Smaller grain size in the 70/30 material could have resulted in scaffolds with greater interconnectivity, thus showing larger shrinkage during sintering and therefore smaller diameters. Furthermore, a higher temperature for sintering β-TCP scaffolds is required compared to bioglass, which is already melting at about 1200 °C. Although the sintering temperature of the 50/50 scaffolds was 100 K higher than the sintering temperature of the 70/30 scaffolds, the higher mass content of β-TCP requires even higher temperatures in the oven. This leads to a lower densification of the scaffolds compared to those with a higher bioglass content. Despite the statistically significant difference in scaffold diameters, the difference remains small at ~5%, and no significant difference in height was detected. 

EDX analysis detected all elements which were expected to be present in the β-TCP and BG composites; calcium, phosphorous, silicon, sodium and oxygen. Hence, silicon is the main component of BG thus considerably increasing the silicon content of the composite material. Differences also exist in the calcium content of the materials. The mean Ca/P ratio for the 50/50 material was 1.86, whereas for the 70/30 material it was 2.03. The increase in Ca/P ratio for the 70/30 material was expected, as BG has a higher Ca/P ratio than β-TCP. As expected, the BG (45S5) showed a Ca/P ratio of 5.27 [28] and the β-TCP a ratio of 1.55 [7]. This information provides an interesting prelude to the immersion in SBF experiment. Based on studies into the effect of Ca/P content on apatite formation, it is expected that by increasing the Ca/P ratio of pure β-TCP with the addition of BG, apatite formation on the material surface should occur when the material is immersed in SBF (see Figure A1 in Appendix). After immersion in SBF for 28 days, a degradation of the samples from 0.61% to 13.67% could be observed. The samples with the highest specific surface area showed the highest degradation. 

3D laser scanning results indicate that the average roughness of the 50/50 scaffolds was higher (Ra 40.11 ± 3.18 µm) than for the 70/30 scaffolds (Ra 38.23 ± 10.25 µm); however, the difference was not hugely significant with only a ~5% increase in the 70/30 Ra. Zareidoost et al. [29] and Li et al. [30] also discovered significantly higher cell growth on samples with higher roughness. High macro- and micro-roughness can increase osteoblast differentiation and hence bone formation on the surface. Based on these results it is expected that cells should successfully proliferate on the surfaces of both samples. A few sinter bridges, where particles have joined together, can be observed; however, many more individual particles are apparent. This poor particle interconnectivity should be noted as it may affect the mechanical strength of the scaffolds. Hence, the study by Vorndran et al. [27] indicated that scaffolds with small, well interconnected particles showed higher mechanical strength than those with larger, individual particles.

Although the mercury porosimetry results reported the total porosity, average pore radius and total cumulative volume to be slightly higher for the 50/50 scaffold than the 70/30 scaffold, for the purpose of this project the differences can be considered negligible. It can therefore be concluded that the scaffolds have been successfully printed with similar porosities and that difference in porosity is not a factor which could influence other experiments carried out using the scaffolds. Lawrence et al. [28] postulated that a porosity is very important for cell colonization in 3D scaffolds. The total porosity of the scaffolds (between 70% and 75%) is in the region of that for many similar publications, although Polo-Corrales et al. [5] believes that a higher porosity is ideal. Moreover, the scaffolds contain a range of macropores, mesopores and micropores which has been proven to encourage osteoblast activity in addition to aiding various types of essential bone growth. In addition to the wide range of macro and microporosity which enhances cell behavior, the large pore channels running throughout the scaffolds should aid supply of essential nutrients and formation of blood vessels. Comparing the mercury porosimetry results to the information on porosity found in literature, the porosity characteristics of the scaffolds are encouraging [5]. Based on this, it is reasonable to expect the cell culture experiments to show cell growth into both types of scaffold material. With reference to the study by Zhang et al. [31] it is possible that incorporating controlled nanoporosity into the scaffolds via nanofabrication techniques could further improve the porosity characteristics of the scaffolds by allowing closer control over the surface porosity with the aim of improving cell adhesion. For a faster osseointegration, the 3D scaffolds could additionally be coated with nanoparticles of growth factors, as already described by Gong et al. [32] and Yi et al. [33]. This would promote bone growth on a nanoscale. The high porosity of the constructs could even serve for a guided bone growth.

The compression tests found the 70/30 scaffolds to be ~13% stronger in compression than the 50/50 scaffolds. This is in-keeping with the fact that BG has superior mechanical properties when compared to β-TCP and therefore the scaffolds with a higher BG content should be stronger in compression. However, both scaffolds had considerably weaker compression strength than the other 3D printed or plotted scaffolds, which showed compression strengths ranging from 7.4 to 11 MPa and of natural bone, which has a compression strength ranging 2–20 MPa (cancellous bone) and 100–200 MPa (cortical bone) [34]. Roohani-Esfahani et al. also reported a 3D plotted scaffold with 50–200 MPa [35]. In contrast to this, the strongest of the scaffold had a compression strength of just 0.171 MPa, which does not compare to even the weakest forms of cancellous bone. Again, referring to Vorndran et al. [27], poor interconnectivity may be related to low mechanical strength. 

A higher concentration of live cells was present on the 70/30 materials and the 50/50 materials than on the scaffolds out of 100% BG or 100% β-TCP. This is particularly obvious in the images captured after seven days. In addition, the concentration of live cells increased over 3, 7 and 10 days. These results indicate that the osteoblast-like MG-63 cells can survive and proliferate on both materials. The assay results also revealed that cells appeared to survive and proliferate with greatest success around the large pore channels in the scaffolds. Karageorgiou et al. [36] and Zhang et al. [31] demonstrated that cell infiltration and distribution within the whole scaffold will greatly affect the overall performance of the resulting construct. The images in Figure 7 show the areas on the scaffolds with the highest concentration of living cells. Overall, the live/dead assay results are an encouraging indication of good biocompatibility in both materials and agree with other extensive studies proving the biocompatibility of both BG and β-TCP [37]

In summary, there was a higher MG-63 cell proliferation on the 70/30 scaffolds, suggesting that greater suitability of this scaffolds in vivo. Moreover, an increase in absorbance on both the 50/50 and 70/30 scaffolds was observed between three and seven days in each of the experiments. This was expected to increase further between 7 and 10 days, as in the MG63 cell control, but, conversely, the results show an increase in absorbance following 10 days of incubation. It is likely that this a result of scaffold dissolution; as the scaffold degrades with time the surface area for cells to attach to and differentiate on is decreased. With this lack of space, overcrowding occurs and it is impossible for cell proliferation to continue. Due to the faster degradation rate of BG, it is likely that the decrease in proliferation will be greater in the 50/50 scaffolds, as they have a higher β-TCP content and should therefore show greater degradation; however further investigation into the solubility of the scaffolds would be necessary to confirm this.

With reference to scaffold behaviour, the results from the LDH assay mainly demonstrate that the 50/50 scaffolds showed a higher LDH activity, than the 70/30 scaffolds. This is demonstrated in Figure 9. Compared to the cell control the LDH activity was lightly increased for 70/30. A higher value could be found for the 50/50. 

Figure 8 also demonstrates the increase in the detected absorption, and therefore cytotoxicity, of the scaffolds with time. Similar to the scaffold degradation discussed in the previous section, as the scaffold dissolves over time more cells will become detached from the scaffold surface and thus will be unable to survive. This increase in cell death with time is detected by the LDH assay as an increase in cytotoxicity, when in fact it could potentially be a result of scaffold degradation. For this reason, it can be concluded that the cytotoxicity reported in the LDH assay may not be reliable and therefore is unsuitable for comparison to similar experiments. Overall, the LDH assay is effective in indicating that the 50/50 material has higher levels of cytotoxicity than the 70/30 scaffold. Given the higher viability of the 70/30 scaffolds indicated in the WST assay, this is the result which was expected. The LDH measurements were made after 1, 2 and 3 days. The WST measurements were made after 3, 7, and 10 days. The higher LDH activity is equivalent to a higher cell death rate at the beginning of the experiment. This results in a low cell count on the scaffolds. Thereafter, the cells show a normal, comparable to the control, albeit less growth. Compared to the cell control, the absorbance of the scaffolds in WST shows smaller values. This is because there are fewer cells on and in the scaffolds than in the cell culture plate, that was used as control. For correct values the absorbance of the scaffolds shown in Figure 8a, should be combined with the absorbance of cells that remained in the cell culture plates in Figure 8b. 

## 4. Materials and Methods

The bioglass (BG) was obtained from Colorobbia, Italy, the β-tricalcium phosphate (β-TCP) from Budenheim, Germany and the dextrin from Carl Roth, Germany. The chemical composition of the bioglass was 47.3 wt % SiO_2_, 28.6 wt % CaO, 15.2 wt % P_2_O_5_, 4.9 wt % Na_2_O, 2.5 wt % MgO and 1.5 wt % F. The scaffolds used in the project were composed of specific combinations of BG and β-TCP scaffolds: 50/50 and 70/30 BG/β-TCP ratios, pure BG and pure β-TCP. As the particle size of the powders was initially too large, it was necessary to mill the powder using grinding balls until the medium particle size was smaller than 2 µm and 90% of the grains were smaller than 5 μm diameter. Moreover, this was suggested to improve the mechanical strength of the scaffolds [21]. The raw materials were milled into a water based suspension containing 65 wt % ceramics. For the BG suspensions, DOLAPIX A88 (Zschimmer & Schwarz, Lahnstein, Germany) was used as dispersant. The suspensions which contained β-TCP were stabilized with DOLAPIX CE64 (Zschimmer & Schwarz, Germany). The suspensions were granulated in a spray dryer (GEA Niro, Gladsaxe, Denmark) at 220 °C using PVA 22000 (Fluka, Buchs, Switzerland) as a binder. The samples were produced with an Inkjet 3D Printer (ZPrinter 310 Plus, Z Corporation, Rock Hill, SC, USA). To increase the green density of the printed scaffolds the powder contained a mixture of a fine (medium diameter at about 5 to 10 µm) and a coarse (medium diameter of the granules at about 35 to 40 µm) fraction. The mass ratio of the coarse to fine powder was 75:25 for all powders except of the 50/50 BG/β-TCP scaffolds, where the mass ratio is 50:50. The powder was also mixed with 10–15 wt % dextrin, a starch based substance which acts as a binder when combined with the water based output from the inkjet (zb60, Z Corporation, Rock Hill, SC, USA), as dextrin is water soluble and its dissolution allows the particles to bind together [38]. The layer thickness was 100 µm and the binder/volume-ratio for the shell and the core was 0.4 and 0.2, respectively. After the binder was dried, the samples were de-powdered and sintered to improve the mechanical properties. The sintering protocol differed for each different material composition used. The sintering temperatures for the pure β-TCP was 1250 °C [39]. The BG and the 50/50 scaffolds were heated to 350 °C and kept at this temperature for 60 min. The temperature was then increased to 950 °C and the scaffolds were then exposed to this temperature for 120 min before cooling down gradually in the oven. The process was similar for the 70/30 scaffolds except that in the second stage of the process they were heated to 1050 °C rather than 950 °C and kept at this temperature for 140 min. A sinter shrinkage of around 10–15% could be observed for the scaffolds. The scaffolds consisting of pure β-TCP were also debindered for 60 min at 350 °C and then heated to 1250 °C, keeping this temperature for 240 min. To avoid cracks during the cooling, the temperature was decreased to 200 °C with a cooling rate of 100 K/h.

### 4.1. Sample Characterization

The ceramics (3 samples of each) were tempered at 105 °C for 24 h to ensure that no water was present in the samples. The test was then carried out in accordance with DIN 66133. For this purpose, the samples were transferred into a glass vessel and this was installed in the Pascal 140/440 porosimeter. The pressure control and evaluation were carried out by PASCAL software. The results from both measurements were then combined into a diagram by the software [40]. The SEM/ESEM images were taken with FEI Quanta 250 FEG (FEI, Hillsborough, OR, USA) and Philips ESEM XL 30 FEG (Amsterdam, The Netherlands). The EDX measurements were carried out on a Philips ESEM XL 30 FEG with EDX unit and the EDX measurements in Appendix A1 were carried out on a FEI Quanta 250 FEG with Oxford EDX unit. The measurement conditions were 10 kV accelerating voltage and 10 min measurement time (live time). A Keyence 3D Laserscanning Microscope (Keyence VK-X210) was used to measure the surface roughness. All experiments were carried out at room temperature. The uniaxial compression tests were carried out at a Zwick/Roell Z005 (Zwick/Roell, Ulm, Germany) materials testing machine with Test Expert II Software. A standard compression test was carried out with 1 N pre force and testing speed of 10 mm/min. Stop criterion was break or 20% length deformation. Origin 2016 Professional (OriginLab, Northampton, MA, USA) was used for all following statistical calculations. 

### 4.2. Cell Culture Experiments

The cell culture experiments revealed the effect of the composite on MG-63 cells (ATCC CRL-1427) via live–dead assays and Giemsa staining. The live–dead cell staining kit II (Art. No. PKCA707-30002, Promokine, Heidelberg, Germany) was obtained from Promokine and the azur eosin methylene blue (Art. No. 109204, Merck, Lucerne, Switzerland) for Giemsa staining was obtained from Merck. To sterilize the 3D printed scaffolds, they were heated up to 200 °C for 4 h in a Drying oven (Memmert, Schwabach, Germany, UN 100). 

#### 4.2.1. Cell Proliferation Assay

The scaffolds for use in the WST experiments were seeded in a 24 well plate. Each experiment required 10 scaffolds of each material composition, with cells seeded in an empty well to act as a control and a blank to account for background absorbance in the ELISA reader. The experiments were repeated three times. The scaffolds and control well were seeded with cell solution containing 50,000 cells in 400 µL. Once seeded, the well plate was incubated at 37 °C, 5% CO_2_ for 2 h and then 1.5 mL DMEM-F12 (Art. No. BE12-719F, Lonza, Basel, Switzerland) was added to each well. The WST experiments were carried out 3, 7 and 10 days following seeding. The same procedure was repeated at each interval: The DMEM-F12 was removed from each well and the scaffolds were washed with DPBS (Art. No. 14190-094, Gibco, Grand Island, NE, USA) three times to remove all medium residues from the pores. DMEM-F12 phenol red free (1 mL) (Art. No. 11039-021, Gibco, Grand Island, NE, USA) was added to each cell-containing well, plus one empty well to act as a blank. WST reagent (100 µL) (Art. No. 05015944001, Roche, Basel, Switzerland) was then added to all of the wells in use. These volumes were sufficient to fully immerse the scaffolds. The well plate was then placed in an incubator at 37 °C, 5% CO_2_ for 2 h. After incubation, three 100 µL samples were taken from each well and placed in a 96 well plate. The plate was placed in a Spectrostar Nano microplate reader (BMG Labtech, Ortenberg, Germany) and subject to 60 s orbital shaking before the absorbance was measured at wavelength λ = 450 nm with a reference λ = 600 nm. For the additional Giemsa stainings (see Appendix) Giemsa’s azur methylene blue solution (Art. No. 1.09204.0500, Merck, Switzerland was used) according to the following protocol: washing the samples with DPBS (3x), fixing with methanol (Art. No. CP43.3 Carl Roth, Karlsruhe, Germany) for 10 min, washing again with DPBS (3x), using the Giemsa solution 1:10 for 10 min followed by washing with water (3x) and air drying. 

#### 4.2.2. Lactate Dehydrogenase (LDH) Assay

The scaffolds for use in the LDH experiment were seeded in a 24 well plate. Each experiment assessed 10 scaffolds from each material composition, a positive and negative control and a blank to account for background absorbance in the ELISA reader. The experiments were repeated three times. A 400 µL cell solution containing 50,000 cells was seeded onto each scaffold and additionally into two empty wells to act as the positive and negative controls, respectively. One well was left empty for use as a blank. The well plate was placed in an incubator at 37 °C, 5% CO_2_ for 2 h. Following incubation, 1.5 mL DMEM-F12 phenol red free was added to each cell-containing well, plus one additional empty well to act as the blank. Triton X-100 (10 µL) (Art. No. X100, Sigma Aldrich, Saint Louis, MO, USA, was added to the positive control wells. The plates were then returned to the incubator. The LDH experiments were carried out at 24, 48 and 72 h following seeding and the same procedure was repeated at each interval: Three 100 µL samples were taken from each well into a 96 well plate. LDH reagent (100 µL) was added to each well in use and the plate was incubated in darkness at room temperature for 30 min. Following incubation, the plate was placed in a Spectrostar Nano microplate reader (BMG Labtech, Ortenberg, Germany) and absorbance was measured at a λ of 490 nm with a reference λ of 600 nm. The measured values were normalised to the cell control (without any 3D printed sample) [26].

#### 4.2.3. Live–Dead Assay

The scaffolds for use in the live/dead assay were seeded in a 24 well plate. For each experiment, at least 10 different samples of each composite/control were used, and the experiments were repeated three times. A 400 µL cell solution containing 50,000 cells was seeded onto each scaffold and the plate was placed in an incubator at 37 °C, 5% CO_2_ for 2 h. Following incubation, 1.5 mL DMEM-F12 was added to each well in use and the plates were then returned to the incubator. The live/dead experiments were carried out at 3, 7 and 10 days following seeding and the same procedure was carried out at each interval: The scaffolds were moved to a new 24 well plate and washed three times with DDPBS to remove any excess medium within the pores. The scaffolds were then cut longitudinally through their centers with a small sterile razor blade. One half of each scaffold was cut again in the transverse direction. One longitudinal and one transverse section of each scaffold was placed in separate new wells. The live/dead solution was prepared, combining 8 mL DDPBS, 16 µL EthD III and 4 µL calcein out of the live/dead cell staining kit II. live/dead solution (1 mL) was added to each scaffold-containing well. The plate was incubated at room temperature for 10 min. Following incubation, an Olympus BX 51 fluorescence microscope with U-MWIB3 broadband blue excitation with interference band-eliminator filter: excitation filter Olympus BP460-495, beam splitter Olympus DM505, band-eliminator filter BA510F was used to view the live and dead cells simultaneously. For automatically cell counting ImageJ (National Institutes of Health, Bethesda, MD, USA)version 1.47v according to Grishagin [41] was used. The cells were counted at 5 different positions of 1 mm² at the same scaffold. This procedure was repeated for all different scaffolds and for at least 5 different samples of each point of interest.

#### 4.2.4. Preparation of Simulated Body Fluid

Simulated body fluid (SBF) (500 mL) was prepared as described by Jalota et al. [42]. The chemicals used to prepare the SBF and the quantities which were added are outlined in Table 7. A beaker of deionised water was placed on a magnetic stirrer at 37 °C. Each chemical was weighed using electronic scales and added to the deionized water in the order presented in Table 8. An electronic pH meter (Mettler Toledo, EL20, Columbus, OH, USA) was then used to measure the exact pH of the solution, and hydrochloric acid was added slowly until the solution reached a pH of 7.4. The beaker was then covered with aluminum foil and left on the magnetic stirrer overnight. The following day, the solution was filtered through a 0.2 µm (pore size) filter and sealed under sterile conditions. At least 3 samples of each were immersed in SBF for 28 days. Before and after the SBF experiment, the samples were weighed with a Sartorius scale. The diameter and height of the scaffolds were measured using Vernier calipers.

### 4.3. Statistics

Data were expressed as mean values ± standard deviation of the mean and analyzed by one-way analysis of variance (ANOVA). The level of statistical significance was set at *p* < 0.05. For statistical calculations, Origin 2016 Professional SR1 (OriginLab) was used. Each Experiment, except the mechanical testing with three samples each, was repeated at least three times with a minimum of 10 samples.

## 5. Conclusions

The results predominantly explore to what extent the fabrication technique is appropriate for development of scaffolds for use in biological applications. The biocompatibility experiments indicated that MG-63 cells could survive and proliferate on both material compositions. However, as indicated by the lower than ideal Ca/P ratio, apatite distribution on the material surface is sparse and it is doubtful that this would be sufficient for the material to form a strong bond with bone. The most striking finding from the characterization experiments is the especially low mechanical strength of the scaffolds in compression. It is clear from the information in the literature that, combined with the laser images showing the poor particle interconnectivity and the increased sinter shrinkage of the 70/30 material, the poor mechanical strength is largely a result of printing process parameters rather than properties of the materials or scaffold geometry. It is unlikely that it will be possible to increase the mechanical strength of scaffolds of this nature to an extent where they would be suitable for use in load bearing applications. However, bioactive degradable scaffolds could prove to be useful in other non-load bearing applications such as acting as fillers in cases of tumor removal or similar applications that require filling a gap in an area which is not subject to high stresses. 

## Figures and Tables

**Table 1:** Dimensions of 3D printed Samples (*n* = 10).

	BG	β-TCP	50/50	70/30
Diameter (mm)	10.19 ± 0.06	10.41 ± 0.25	11.09 ± 0.02	10.60 ± 0.02
Height (mm)	2.88 ± 0.10	2.45 ± 0.16	2.84 ± 0.12	2.94 ± 0.26
Weight (mg)	251.92 ± 12.90	190.30 ± 7.13	223.85 ± 15.56	238.67 ± 18.91

**Table 2:** Mean elemental composition for the samples (ZAF corrected).

Material	BG	β-TCP	50/50	70/30
Element	Atom %	Atom %	Atom %	Atom %
F_2_O **	2.8	0	2.0	2.5
Na_2_O	3.8	0	1.7	2.6
MgO	4.8	0	3.8	5.3
Al_2_O_3_	0.8	0	0.8	1.0
SiO_2_	41.5	0	18.8	27.1
PO_4_	7.4	39.1	25.5	20.3
CaO	38.9	60.9	47.4	41.2
**Ca/P ratio**	**5.3**	**1.5**	**1.9**	**2.0**

Philips ESEM XL 30 with EDX unit was used for the integral measurement, 10 min (live time), area 0.81 mm^2^; ** only for Calibration purposes.

**Table 3:** Surface Roughness of the 3D printed Scaffolds (selected parameters) (*n* = 10).

Material	BG		β-TCP		50/50		70/30	
Parameter	Rz (µm)	Ra (µm)	Rz (µm)	Ra (µm)	Rz (µm)	Ra (µm)	Rz (µm)	Ra (µm)
Mean	78.33	11.82	207.29	26.08	183.81	28.92	194.50	27.61
SD	9.17	1.58	37.69	7.01	8.64	4.71	38.62	8.86
% RSD	3.24	0.56	13.33	2.48	3.06	1.66	13.66	3.13

**Table 4:** Mercury Porosimetry parameters for the scaffolds (*n* = 3).

Material	BG	β-TCP	50/50	70/30
Total cumulative volume (mm³/g)	232.50	319.62	788.18	713.91
Total specific surface area (m²/g)	0.09	0.06	1.08	1.19
Average pore radius (µm)	5.84	11.99	10.57	9.58
Total porosity (%)	46.99	63.73	74.12	71.29
Bulk density (g/cm³)	2.02	1.99	0.94	0.99
Apparent density (g/cm³)	3.81	5.49	3.63	3.48
Sample volume correction	0.98	none	0.95	0.95

**Table 5:** Failure load and compressive strength of the 3D printed Scaffolds (*n* = 3).

Sample	BG	β-TCP	50/50	70/30
**Failure load (N)**	51.90 ± 17.10	54.30 ± 14.50	14.30 ± 1.10	15.10 ± 1.10
**Compressive strength (MPa)**	0.64 ± 0.21	0.64 ± 0.17	0.15 ± 0.01	0.17 ± 0.01

**Table 6:** Comparison of living and dead cells per mm² on the different materials.

Time	BG		TCP		5050		7030	
	Cells/mm²		Cells/mm²		Cells/mm²		Cells/mm²	
	Alive	Dead	Alive	Dead	Alive	Dead	Alive	Dead
3 days								
Outer surface	80 ± 9	7 ± 2	68 ± 7	5 ± 1	79 ± 8	6 ± 1	70 ± 5	5 ± 2
Inner surface	208 ± 44	6 ± 4	321 ± 42	6 ± 3	119 ± 42	3 ± 1	72 ± 13	8 ± 4
7 days								
Outer surface	634 ± 48	62 ± 14	126 ± 3	18 ± 3	480 ± 17	52 ± 22	576 ± 119	74 ± 4
Inner surface	197 ± 15	32 ± 23	191 ± 14	4 ± 1	164 ± 17	16 ± 11	178 ± 2	24 ± 3
10 days								
Outer surface	670 ± 167	0	636 ± 63	8	516 ± 28	10 ± 3	534 ± 31	10 ± 3
Inner surface	277 ± 32	0	278 ± 11	0	289 ± 72	0	251 ± 20	0

**Table 7:** Degradation of 3D printed Samples after immersion in SBF for 28 days (*n* = 3).

	BG	β-TCP	50/50	70/30
∆ Diameter (%)	0	1.96 ± 0.03	1.98 ± 0.25	2.36 ± 0.56
∆ Height (%)	0.54 ± 0.05	0	0.12 ± 0.02	0.24 ± 0.12
∆ Weight (%)	8.08 ± 0.97	0.61 ± 0.03	12.03 ± 3.63	13.67 ± 4.91

**Table 8:** Composition of Tris-buffered SBF 27.

Reagent	Obtained by	Art. No.	Quantity (g)
Sodium chloride (NaCl)	Roth	3957.3	3.274
Sodium hydrogen carbonate (NaHCO_3_)	Roth	6885.2	1.134
Potassium chloride (KCl)	Sigma	P5405	0.187
di-sodium hydrogen phosphate dihydrate (Na_2_HPO_4_·2H_2_O)	Merck	6580.0500	0.089
magnesium chloride hexahydrate (MgCl_2_·6H_2_O)	Sigma	M8266	0.071
calcium chloride dihydrate (CaCl_2_·2H_2_O)	Sigma	C7902	0.184
sodium sulphate (Na_2_SO_4_)	Sigma-Aldrich	746363	0.0355
Tris ((CH_2_OH)_3_CNH_2_)	Serva	37180	3.0285
1M HCl	Roth	CN63.1	Until pH 7.4

**Figure A2:**
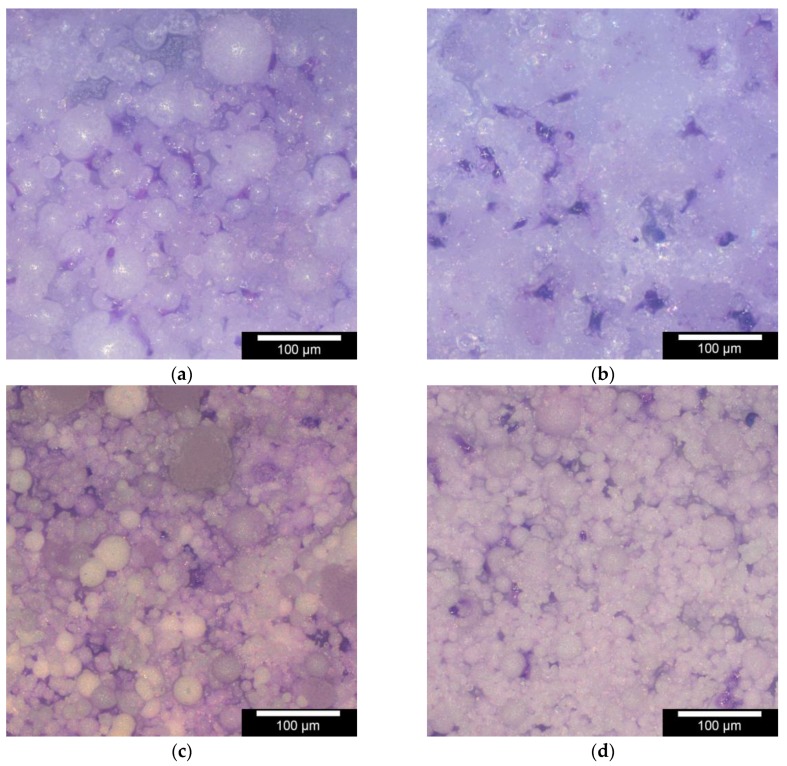
Giemsa Staining of MG63 cells on the Scaffolds after 24 h: (**a**) BG; (**b**) TCP; (**c**) 50/50; and (**d**) 70/30: Images taken with Olympus BX 51 with reflected light in 10× magnification. The staining was according to the protocol given in Romeis [43].

## Appendix A

Because of the 3D structure of the samples, the Giemsa staining was an alternative to show the cell attachement on the ceramics. As shown in Figure 3 and Figure 4, the surface of the samples is not flat. Thus, the The cell core is stained in violet and the cytoplasm dark blue. The images were taken with Olympus BX 51 Light microscope.
